# Terahertz Nonlinear Ghost Imaging via Plane Decomposition: Toward Near-Field Micro-Volumetry

**DOI:** 10.1021/acsphotonics.2c01727

**Published:** 2023-03-10

**Authors:** Luana Olivieri, Luke Peters, Vittorio Cecconi, Antonio Cutrona, Maxwell Rowley, Juan Sebastian Totero Gongora, Alessia Pasquazi, Marco Peccianti

**Affiliations:** † Emergent Photonics Research Centre, Department of Physics, Loughborough University, Loughborough LE11 3TU, UK; ‡ Emergent Photonics Lab (Epic), Department of Physics and Astronomy, University of Sussex, Brighton BN1 9QH, UK

**Keywords:** hyperspectral imaging, ghost imaging, volumetry, terahertz imaging, 3D imaging

## Abstract

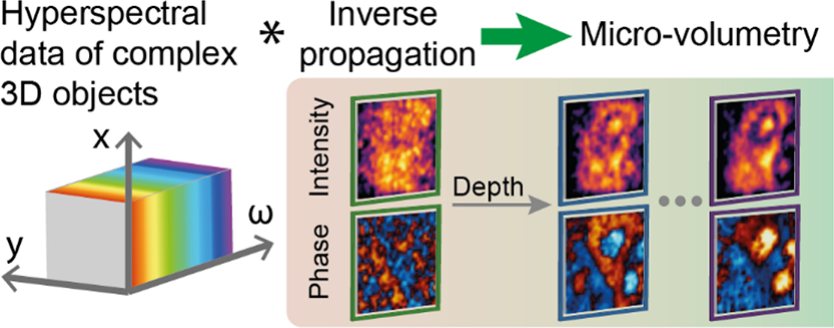

Terahertz time-domain imaging targets the reconstruction of the full electromagnetic morphology of an object. In this spectral range, the near-field propagation strongly affects the information in the space–time domain in items with microscopic features. While this often represents a challenge, as the information needs to be disentangled to obtain high image fidelity, here, we show that such a phenomenon can enable three-dimensional microscopy. Specifically, we investigate the capability of the time-resolved nonlinear ghost imaging methodology to implement field-sensitive micro-volumetry by plane decomposition. We leverage the temporally resolved, field-sensitive detection to “refocus” an image plane at an arbitrary distance from the source, which defines the near-field condition, and within a microscopic sample. Since space–time coupling rapidly evolves and diffuses within subwavelength length scales, our technique can separate and discriminate the information originating from different planes at different depths. Our approach is particularly suitable for objects with sparse micrometric details. Building upon this principle, we demonstrate complex, time-domain volumetry resolving internal object planes with subwavelength resolution, discussing the range of applicability of our technique.

## Introduction

Diagnostic techniques based on terahertz (THz) electromagnetic radiation attract significant attention because many materials of interest possess unique signatures within the 0.1–10 THz frequency range.^1−3^ THz imaging has been employed to reveal the properties of living cells, human skin,^4−6^ hidden details in ancient manuscripts and paintings,^7,8^ cancerous tissue,^9−12^ and illicit drugs.^13^ In particular, three-dimensional (3D) imaging, e.g., volumetric imaging^14^ or tomography,^15^ has been a long-standing goal for noninvasive analysis with applications from biology to materials science and security. In this framework, volumetric imaging using THz waves has been recently demonstrated via different approaches, including holographic methods and time-of-flight imaging.^16−19^

Regardless of how revealing the THz spectra might be, the relatively long THz wavelength implies that any submillimeter feature of an object is practically invisible to standard diffraction-limited imaging methodologies. Among the recent approaches for THz microscopy, imaging concepts based on spatially structured THz near-field illumination, i.e., “ghost” imaging,^20−22^ demonstrated real-time acquisition,^23^ micrometric resolution, and a high signal-to-noise ratio^24−28^ for thin opaque samples. These approaches rely on illuminating the sample in the near field with a sequence of predetermined spatial patterns and detecting the average scattered field. The patterns are then correlated with the detected signal to reconstruct a spatiotemporal image of the sample. By leveraging optical-to-THz nonlinear conversion, these methods can employ deep subwavelength sampling to increase the achievable spatial resolution drastically. The THz pattern source effectively acts as the probe of the imager, and since the detection happens in the far field, the distance between the THz pattern source and the object defines the near-field condition of the system. In the subwavelength regime, the near-field propagator (i.e., the electromagnetic Green’s function) rapidly varies within the wavelength spatiotemporal scale. As a result, the spatial and temporal information become entangled as the field propagates away from the source. Any subwavelength distance between sources and object planes drastically affects the retrieved image. Consequently, for samples with a complex 3D internal structure, understanding the spectral fingerprint of microscopic features with fidelity, especially for semitransparent objects, is a challenging task.^29−31^

However, the spatiotemporal information of the sample is “scrambled” but not necessarily lost. The development of reconstruction protocols is, hence, critical for reliably interpreting the hyperspectral image, even in the case of simple planar objects in the subwavelength regime. Indeed, the aberrations or errors encountered with methodologies that do not compensate for the spatiotemporal mixing are challenging to identify. For instance, they retain the impression of high spatial resolution but alter the spatial and spectral response in a complex fashion, an effect that is very dissimilar to the loss of resolution (i.e., blurring) occurring in numerical-aperture-limited systems.

We recently addressed a seminal part of this challenging scenario by introducing the time-resolved nonlinear ghost imaging (TNGI)^25,26^ technique. In the TNGI, an ultrafast structured optical beam locally generates the subwavelength THz patterns by a second-order nonlinear conversion. A time-domain spectroscopy (TDS)^32−34^ detection system collects the temporal dynamics of the average scattered field. A computational protocol allows the high-fidelity reconstruction of hyperspectral (in space and time) features.^26^ In the first instance, the TNGI methodology works for planar samples placed in close contact with the generation crystals without any post-processing requirements. Critically, our methodology also enables the reconstruction of planar objects located significantly distant (i.e., well beyond the THz near-field region) from the source plane. This result is achieved by employing a space–time deconvolution methodology to translate the imaging plane at an arbitrary distance from the source. Effectively, our inverse propagator methodology acts as a virtual “refocusing” operator in analogy with standard diffraction-limited imaging systems.

An open question is whether the ability to reconstruct arbitrary planes away from the source can enable volumetric microscopy of 3D samples. Intuitively, the intrinsic spatiotemporal coupling in the subwavelength regime could allow isolating the contribution of the different planes within the object. In an interesting parallel, a rapidly varying point-spread function is routinely exploited in confocal optical microscopy to amplify the spatial separation between planes at different depths of an object. Such a property effectively introduces a rapid blurring of the intensity distribution of out-of-focus planes,^35^ fundamentally enabling the 3D perception of a microscopic sample.^36,37^ Also, state-of-the-art, fluorescence-based volumetric microscopy systems (e.g., SPIM^38^ or SCAPE^39^) rely on the ability to isolate each emission plane by acting on the cross section of the illuminating beam. Interestingly, in our scenario, the near-field electromagnetic space–time coupling possesses similarly rapidly varying features at the subwavelength scale.

In this work, we demonstrate volumetric microscopy of semitransparent objects via TNGI. We leverage an inverse propagator methodology that acts as an equivalent refocusing tool. We show that it is suitable to reconstruct the microscopic morphological features of the sample while locating them in 3D space with subwavelength resolution. Critically, in the TNGI, the spatiotemporal coupling fundamentally enables the perception of the depth dimension. We apply this principle to simple experimental geometries, demonstrating the ability to reconstruct different internal planes of semitransparent objects. Furthermore, we discuss the problem of out-of-focus contributions of nearby sample planes leaking into the selected plane, i.e., the degree of isolation between different planes, providing a criterion to assess the validity of our technique. Our results pave the way for the generalization of the approach toward more complex geometries and the definition of a high-resolution, fully volumetric hyperspectral imaging system.

## Methods

**Figure 1:**
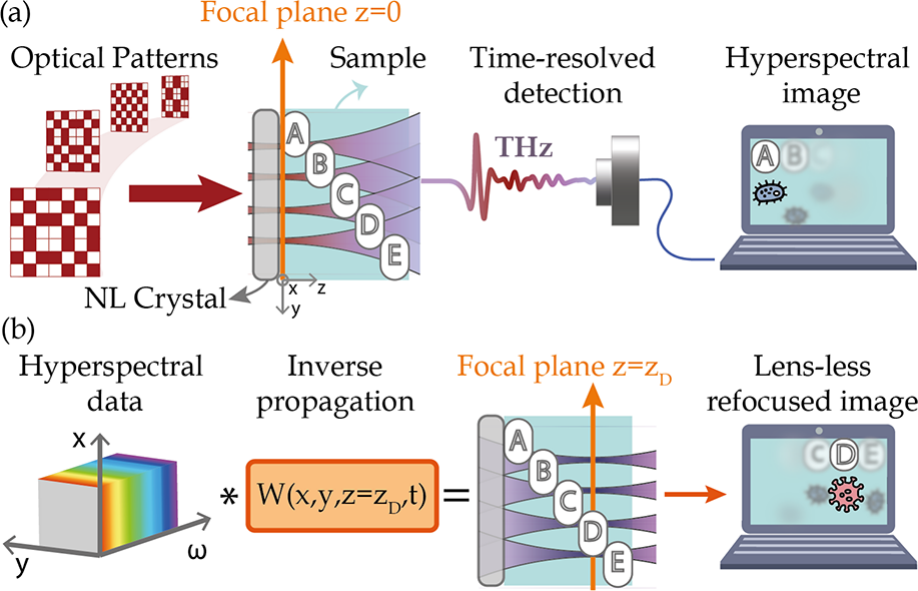
Conceptual overview of 3D hyperspectral imaging via a TNGI approach. (a) Conceptual illustration of the TNGI imaging acquisition. A sequence of optical patterns is nonlinearly (NL) converted to subwavelength THz structured fields, which illuminate the object. The time-resolved average scattered field is collected via a single-element TDS detection. In the presence of a sample with a complex 3D structure, the TNGI reconstructs the imaging plane closer to the source without any post-processing of the spatiotemporal data. (b) Volumetric reconstruction based on an inverse propagator technique. The information encoded in the hyperspectral data is analyzed via an inverse propagation operator *W*(*x*, *y*, *z* = *z_D_*, *t*) that acts as a virtual refocusing tool. As a result, the volumetric features located at different depths *z* of the sample, corresponding to different distances from the source plane, are recovered with high fidelity.

Figure 1 summarizes our approach, with panel (a) reporting the schematic of our TNGI system.^25,26^ A full description of the setup scheme is reported in Figure S1. We implement the time-resolved imaging of a multiplane composite object near a THz source. Specifically, we generate the broadband THz pulse via optical rectification in a 1 mm-thick zinc telluride (ZnTe). We illuminate the crystal with ultrafast pulses derived from a 1 kHz, 100 fs-class regenerative source centered at wavelength λ = 800 nm. We spatially structure the optical source with a digital micromirror array device (DMD), and the micrometric features of the optical patterns are imprinted in the generated THz field. The resulting THz field distribution has a deeply subwavelength spatial structure, and it is near-field-coupled to the sample by placing the latter in direct proximity (i.e., in contact) with the generation crystal. The spatial average of the field scattered by the object is detected at the center of the Fourier plane, which is obtained via a narrow-numerical aperture optical system.^26,27^ The field is collected in time via a nonlinear electro-optic sampler (TDS) and correlated with the standard ghost imaging approach, obtaining a full spatiotemporal function.

In the presence of a 3D sample, the hyperspectral image retrieved via TNGI without any post-processing corresponds to the sample surface adjacent to the THz-generating crystal, as illustrated in Figure 1a. Any part of the object located at a significant distance from the generation crystal is hardly distinguishable due to the strong near-field propagation of the THz patterns.

Here, we post-process the spatiotemporal data to progressively reconstruct the inner planes of an object characterized by a 3D morphology. To illustrate the reconstruction process, we first summarize the TNGI reconstruction of a planar object located at a distance *z* = *z*_0_ from the generating plane, corresponding to the output facet of the nonlinear crystal placed at *z* = 0.^25,26^ The real-valued transmission function *m*(*x*, *y*, *z*_0_, *t*), and its corresponding complex-valued Fourier transform in the frequency space, *M*(*x*, *y*, *z*_0_, ω), contains the spectral and morphological features of the sample. Here, (*x*, *y*) are the spatial coordinates on the plane, *t* is the time, and we consider a frequency-dependent response with the frequency ω. For simplicity, we assume that *M*(*x*, *y*, *z*_0_, ω) is a scalar function with an infinitesimal thickness around the plane *z* = *z*_0_, such that the transmission *A*_out_ of a generic input field *A*_in_ reads as follows:
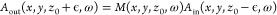
1

In the following, we will omit for simplicity the infinitesimal terms ±ϵ, identifying the sample planes immediately before and after the object. In eq 1, the field *A* is the vector potential of the electromagnetic field. This position allows using rigorously the subsequent scalar analysis for a fully vectorial problem. The electric field *E* (see the Supporting Information) relates to *A* with the Lorentz gauge,^40^ which reduces to a direct proportionality in the first-order approximation. In simple terms, hence, the following discussion can be understood by viewing *A* as equivalent to *E*. The object is illuminated by a set of THz patterns, with field distribution *A_n_*(*x*, *y*, *z*, ω) for the *n*th pattern, generated with a linearly polarized source along *ŷ*, i.e., *A_n_* = *A_n_ŷ*. As we generate the THz field via optical rectification, we can express the electric field *A_n_* as directly proportional to the intensity profile of the optical pattern *I_n_*(*x*, *y*) and to the Fourier transform of the THz electric field *F*(ω), which results from the quadratic rectification of the pulsed optical source:^25^
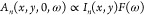
2

It is important to notice that the source is placed on the plane *z* = 0, while the field interacting with the object in eq 1 is at the coordinate *z*_0_. In the simplest case of a homogeneous object or a vacuum, we express such a field through a Green function *G*(*x*, *y*, *z*, *t*) as follows:
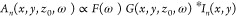
3where the operator * represents a spatial convolution in the transverse coordinates (*x*, *y*). Although the Green function is generally fully vectorial, the problem is scalar in our geometry (see the Supporting Information).

The field detection in the center of the Fourier plane retrieves the average electric field of the transmitted pattern by the object. The TDS is generally a polarization-sensitive technique and can detect the two planar components independently on the (*x*, *y*) plane. As typical in TDS settings, we generate along *ŷ* and detect the field along the same polarization direction. The detected signal for the *n*th pattern is *c_n_*(*t*), whose Fourier transform expressed in the frequency space reads as follows:
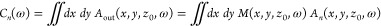
4

We then apply a standard ghost imaging algorithm reconstruction to obtain our spatiotemporal function. Specifically, we employ a predetermined set of orthogonal patterns *I_n_*(*x*, *y*) corresponding to a complete set.^41^ The spatiotemporal reconstruction of the object reads as follows:
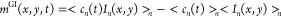
5where <...>_*n*_ denotes the average over the entire distribution of patterns *I_n_*(*x*, *y*) and *c_n_*(*t*) denotes the inverse Fourier transform of eq 4. Thanks to the orthogonality of the incident patterns, the reconstructed function is directly related to the object by the Green function. As discussed in refs (25, 26) and in the Supporting Information, by defining the inverse propagator *W*(*x*, *y*, *z*_0_, *t*) as the inverse of the Green function *G*(*x*, *y*, *z*_0_, *t*) for the given input spectrum *F*(ω), one can retrieve the object with the simple operation
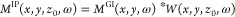
6where *M*^GI^(*x*, *y*, ω) is the Fourier transform of eq 5 and *M*^IP^(*x*, *y*, *z*_0_, ω) is the inverse propagated transfer function, an estimate of *M*. As in eq 3, the convolution operator * acts only in (*x*, *y*). Since the reconstructed plane moves within the object, the coordinate *z*_0_ effectively takes into consideration the optical path within the sample, accounting for the refractive index variations of the material. In the presence of a complex 3D object, however, the field impinging on a feature located at *z* = *z*_0_ cannot be expressed through the Green Function formalism in eq 3. On the contrary, the impinging field will be affected by the interaction with the previous layers of the sample, potentially leading to spurious reconstruction errors and aberrations once the inverse propagation is applied to the hyperspectral data.

To further investigate this scenario and assess the limits of the inverse propagation reconstruction as expressed by eq 6, we fabricated a set of samples characterized by features located at different depths but in well-distinguished points of the (*x*, *y*) plane. After performing a full TNGI reconstruction of the sample, we then applied eq 6 for different values of the coordinate *z*_0_ to reconstruct specific sections of the object (see Figure 1b).

While our technique is clearly affected by the transparency and properties of the first illuminated layers, in principle, it enables significant preservation of the spectral components of distant elements within a single 3D object in a way that is not generally possible in near-field approaches, which do not collect the full temporal information.

Most importantly, our technique relies on the possibility of separating the contributions from the different object planes along *z*. In this framework, the THz subwavelength scenario *dx* ≪ λ, where *dx* is the pixel size, is not only relevant, but it raises unique advantages. The near-field point-spread function *G*(*x*, *y*, *z*_0_, ω), in fact, drastically affects the propagation of the pattern *A_n_*, and each plane of the sample experiences a very different spatiotemporal sampling distribution. As such, the propagation can create sufficient orthogonality between the object morphologies in different planes to allow their separate retrieval.

## Results

**Figure 2:**
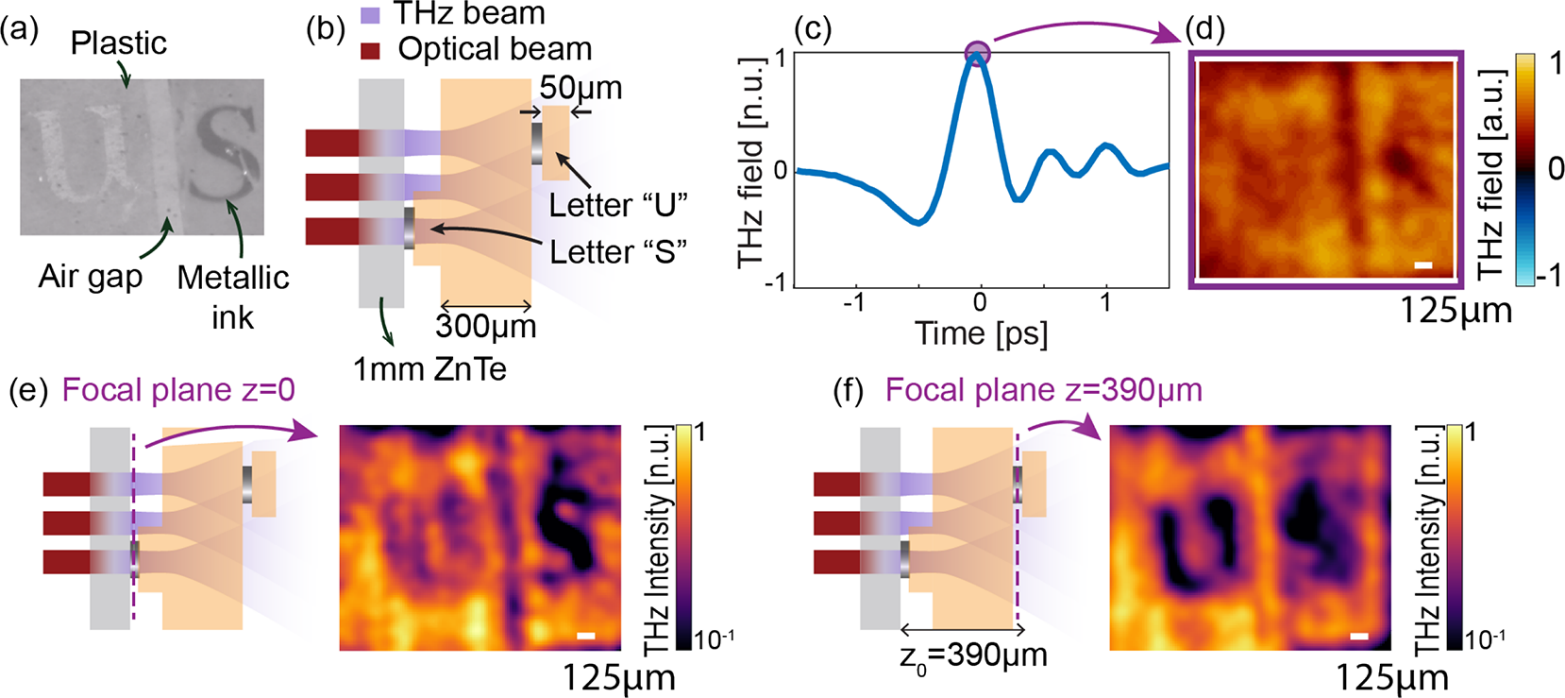
Volumetric reconstruction of a semitransparent object with subwavelength metallic features. (a) Optical image of the sample: metallic letters “U” and “S” on plastic substrates. (b) Near-field imaging scheme and 3D structure of the sample. The sample is placed in close proximity to the nonlinear crystal and is composed of two metallic letters, each deposited on a 50 μm-thick substrate (Kapton) and separated by a 300 μm-thick plastic slab, leading to an overall distance between the two letters of ∼350 μm. (c) Temporal response of the sample, corresponding to the spatial average of the collected function, *m*^GI^(*x*, *y*, *t*). (d) Fixed-time image *m*^GI^(*x*, *y*, *t*_max_) of the sample in correspondence with the peak time *t*_max_. (e) Hyperspectral image *M*^GI^(*x*, *y*, ω), averaged between 1.6 and 2.3 THz, without any inverse propagation operator. In this scenario, the TNGI directly reconstructs the parts of the sample closer to the generating crystal (f) same as panel (e) but following the application of the inverse operator with *z*_0_ = 390 μm. In all the panels, the total field of view was 2 mm × 2 mm with a 32 × 32 spatial sampling. Supplementary videos are reported for the raw data (Videos S1 and S2).

As a first case study, we experimentally investigated the reconstruction of a multilayer 3D object. The sample was composed of two thin metallic objects with subwavelength features corresponding to the letters “U” and “S” (Figure 2).

The metallic letters have an approximate footprint of 800 μm × 800 μm. These two layers were placed at different depths and separated by a thick plastic substrate. The total distance between the two metallic layers was ∼350 μm. We optimized the sample geometry to study the separability of the two planes containing subwavelength morphological features placed at a distance comparable with the carrier wavelength of the THz illumination (λ = 300 μm). The sample was placed directly on the nonlinear generating crystal so that the first metallic mask (“S” letter) was located on the source plane. In our imaging experiments, the total field of view was 2 mm × 2 mm in the transverse direction, sampled with a complete set of orthogonal THz patterns composed of 32 × 32 pixels. Specifically, we employed a complete set of Walsh–Hadamard patterns (with “Russian Dolls” ordering^41^), which is known to maximize the signal-to-noise ratio (SNR) in experiments.^42^ For each illumination pattern, we collected the corresponding full TDS waveform at the center of the Fourier plane and reconstructed the 3D function *m*^GI^(*x*, *y*, *t*) following eq 5. Figure 2c reports the spatial average ∬*m*^GI^(*x*, *y*, *t*)*dxdy* of the experimental spatiotemporal image, while Figure 2d illustrates the spatial distribution *m*^GI^(*x*, *y*, *t*_0_) of the reconstructed field at the peak time *t*_0_ = *t*_max_ = 0 ps. While no specific morphology is easily detectable in the fixed-time reconstruction, the object morphology can be clearly seen in the frequency domain, in close analogy with previous results.^26^ Such a result is illustrated in Figure 2e, where we report the spatial distribution of *M*^GI^(*x*, *y*, ω) averaged between 1.6 and 2.3 THz. When no inverse propagation is applied, the system directly reconstructs the object plane closer to the source surface. In this case, the hyperspectral image *M*^GI^(*x*, *y*, ω) clearly shows the image of the letter “S”, located on the reference plane *z* = 0. The letter “U”, conversely, can be barely distinguished because of the spatiotemporal coupling affecting the THz patterns.

To reconstruct the volumetric information at different planes, we applied the inverse operation defined in eq 6, spanning a set of depth *z*_0_ to maximize the visibility of the letter “U”. Figure 2f illustrates the virtual refocusing of our experimental hyperspectral image, leading to the reconstruction of the second metallic letter at a depth coordinate *z*_0_ = 390 μm. Such a distance corresponds to the overall spacer thickness of the plastic substrate (∼350 μm, *n* ∼1.6), some air gaps between the sample and the nonlinear crystal, and glue among the layers of the samples.

As the letters in our sample were relatively far from each other, the TNGI allowed separating the two contributions relatively easily. Notably, the image at the plane *z* = 0 in Figure 2e was unaffected by the letter “U”, showing that the spatiotemporal coupling significantly reduced the correlation between planes placed at a wavelength distance. Once we refocused the hyperspectral data on the letter “U” along the depth, we still observed a distorted shadow of the letter “S” (Figure 2f), likely due to the absorption of the light at the input plane. Although this result suggests that a fraction of the spectral fingerprint of an occluded object is not immediately accessible with this methodology, the influence of the out-of-focus plane of the letter S appears diminished. Hence, an important question is whether imaging the occluded regions of a target is achievable in semitransparent objects, which are less prone to information loss by absorption.

### Volumetric Reconstruction of Teflon and Sugar Particles in a Complex 3D Structure

**Figure 3:**
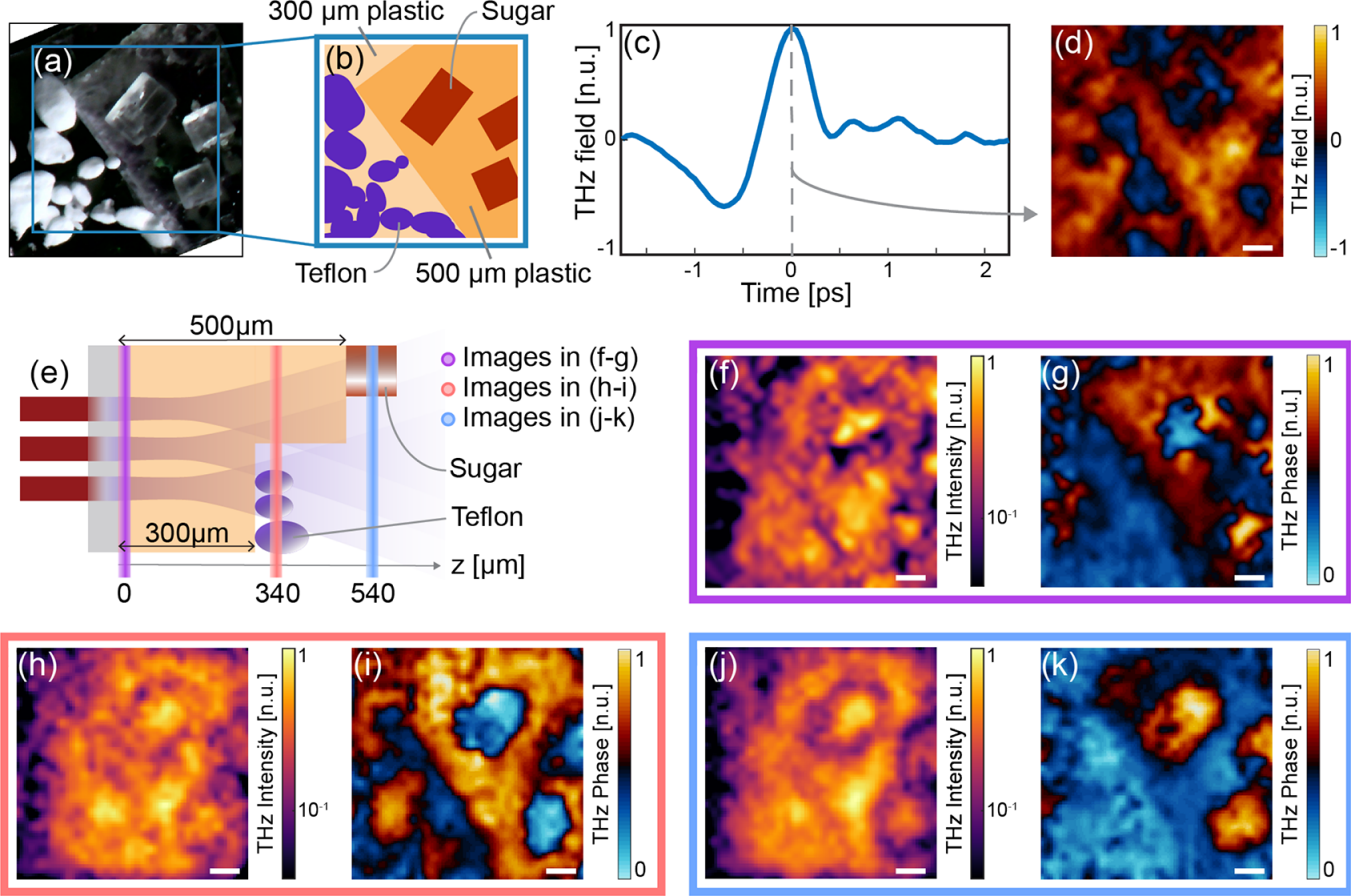
Volumetric reconstruction of a complex 3D object via refocusing: Teflon and sugar particles on plastic slabs. (a) Optical image of the sample. (b) Conceptual illustration of the sample’s composition. (c) Temporal response of the sample. (d) Fixed-time reconstructed images at the peak time. (e) Conceptual sketch of the imaging scheme: refocusing planes highlighted. (f, g) Spectral and phase images at 0.5 THz at the initial focal plane *z* = 0 μm. (h, i) Spectral and phase images at 0.5 THz averaged between *z* = 310 μm and *z* = 370 μm, showing Teflon’s contribution. (j, k) Spectral and phase images at 0.5 THz averaged between *z* = 510 μm and *z* = 570 μm, highlighting the sugar’s contribution. In all panels, the field of view is 4 mm × 4 mm with a 32 × 32 spatial sampling. The white bar represents the 500 μm scale. Supplementary videos are reported for the raw data (Videos S3 and S4).

To address this question, we fabricated a thick multilayer sample composed of two superimposed plastic slabs (with a thickness of 300 and 200 μm, respectively). On top of each slab, we deposited Teflon and sugar particles, as illustrated in Figure 3a,b. Figure 3e provides a cross section of the sample for further clarity.

As a first step, we performed a TNGI analysis with the same setup and experimental parameters as in the previous case. The time-resolved spatial average of the spatiotemporal image *m*^GI^(*x*, *y*, *t*) and the fixed-time image at the peak field (i.e., for *t*_0_ = 0) are reported in Figure 3c,d. Similar to the previous case (cf. Figure 2d), the fixed-time image is difficult to interpret, and all the different components of the sample appear almost indistinguishable in this image.

As the plastic slab placed close to the nonlinear crystal has no morphological features, even the information found in the frequency domain is hard to interpret, as illustrated in Figure 3f,g, where we report the amplitude (panel f) and phase (panel g) of the hyperspectral image *M*^GI^(*x*, *y*, ω_0_) for ω_0_ = 0.5 THz. We remark that these results correspond to the TNGI reconstruction of the sample at the source plane (*z*_0_ = 0 μm) without any inverse propagation operation. Quite interestingly, while the morphology of the particles is not easily detectable, the hyperspectral phase clearly shows the difference in thickness between the two plastic slabs composing the object.

Even for this sample, the inverse propagation analysis allows changing the focal plane and scanning the sample properties along the depth direction. In this scenario, we observed two sets of main features at *z*_0_ = 340 μm (Figure 3h,i) and *z*_0_ = 540 μm (Figure 3j,k). The former image (*z*_0_ = 340 μm) clearly shows the presence of the Teflon particles, evident in the hyperspectral phase reconstruction (Figure 3i). Teflon is a weakly absorbing material at this frequency, and it mostly contributed to a phase shift. These features appeared superimposed on the phase contrast due to the different thicknesses of the plastic substrates. As shown in Figure S2, where we provide a full hyperspectral study of Teflon particles, the spectral response indeed consisted almost entirely of a phase contribution. At the same time, it did not show a strong absorption or edge scattering that could be visible via a THz amplitude image. Similarly, around *z*_0_ = 540 μm, the sugar particles were visible in both amplitude and phase images, and a detailed hyperspectral analysis of this case is included in Figure S3. Remarkably, the images in Figure 3j,k were refocused on a plane behind the Teflon region, but the morphology of the Teflon particles was practically absent both in amplitude and phase. This observation strongly suggests that, in the absence of absorption, the correlation of subwavelength details was rapidly lost in propagation, potentially enabling the imaging of occluded features.

## Discussion: Isolating Planes in TNGI

The ability to locate and precisely extract the contributions of a species along the thickness axis depends on a few practical considerations. First, THz time-domain detection grants access to sub-cycle temporal resolutions; quantitatively, temporal steps down to *T*/1000, where *T*∼2 – 5 ps, are feasible with off-the-shelf technologies. As such, we expect that temporal resolution does not play a significant role in plane discrimination.

In terms of sample properties, we observe that the detected hyperspectral image *M*^GI^(*x*, *y*, ω) is a 3D scalar matrix, while the target function *M*(*x*, *y*, *z*, ω) is, in general, defined in a four-dimensional space. Our approach does not have direct access to the full depth dimension; hence, a full four-dimensional detection of the object morphology function lies beyond the scope of this work. Our methodology aims to develop a way of interpreting the information contained in *M*^GI^(*x*, *y*, ω) in a meaningful way by locating the source of scattering in the depth coordinate. Such a proposition works as long as we can discern the contribution of the scattering points at different depths. Specifically, when applied to the TNGI, our inverse propagation technique enables us to refocus on a specific plane within the sample by taking advantage of the rapidly varying point-spread function (PSF) experienced by the THz probing beam. The rapidly varying PSF fundamentally couples the detected spatiotemporal function *M*^GI^(*x*, *y*, ω) to the object’s internal planes.

To visualize such an effect, we numerically investigated the propagation effects of different subwavelength features for different characteristic sizes. More specifically, we compute, via the Green function,^40^ a feature’s projected dimension onto adjacent planes at different depth coordinates. By decomposing the images in a series of harmonics via Fourier transformation, we can discuss how each spatial frequency propagates to understand the spatiotemporal problem.

**Figure 4:**
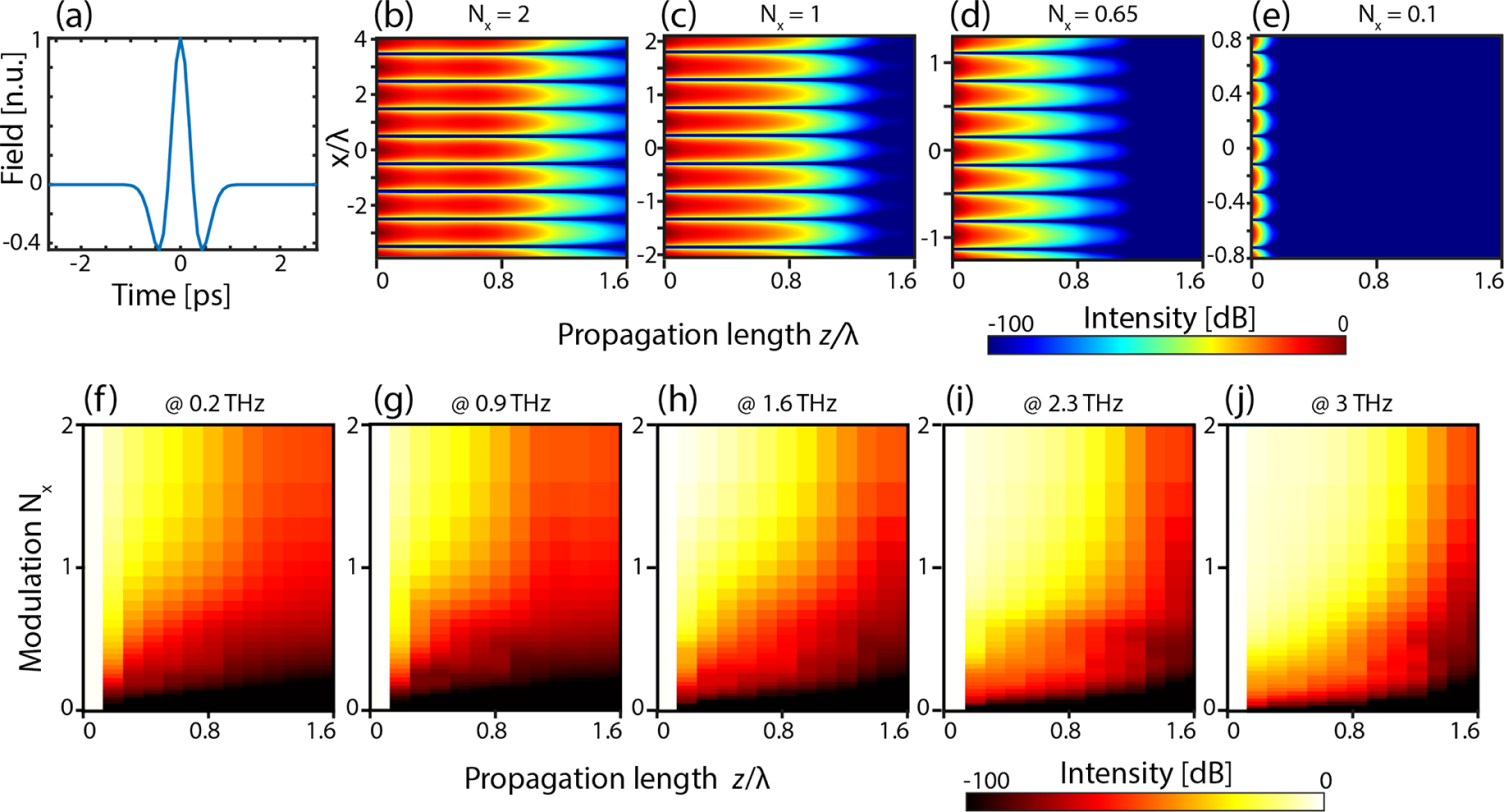
Propagation of a THz spatial-spectral component. (a) Simulated temporal response of the generated THz field. (b–e) Intensity plot showing the propagation of a spatial modulation with scaling factor *N_x_* along the *z* direction. The spectral frequency is 1 THz. (f–j) Evolution of spatial spectrum intensity of the modulation along *z* for different carrier frequencies.

In our physical settings, the THz wave is generated through eq 2, and we can safely assume that the incident field is expressed as
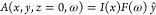
7where *I*(*x*) is the transverse spatial coordinate and *F*(ω) is the THz pulse spectrum. We assumed that the incident THz pulse has a temporal profile *f*(*t*) = (1 – 2(*t*/Δ*t*_0_)^2^)*e*^–(*t*/Δ*t*_0_)^2^^, and its corresponding Fourier transform *F*(ω) employed in the simulations is reported in Figure 4a. This function is the standard pulse resulting from the rectification of an optical Gaussian pulse with Δ*t*_0_ = 350 fs (around 250 fs Gaussian width σ) compatible with our experimental setting, resulting in a spectrum centered at about 1 THz. We assumed a one-dimensional transverse spatial distribution for simplicity. As a spatial profile, we consider a spatial harmonic modulation along *x*, *I*(*x*) = cos (2π*kx*) with *k* = *N*_*x*_^–1^λ^–1^, where λ = 335 μm is the length of the order of the carrier wavelength of the pulse and *N_x_* is a scaling factor that we use to distinguish subwavelength (*N_x_* < 1) to superwavelength features (*N_x_* > 1).

Figure 4 shows the results of our simulations, where we analyzed the propagation of different patterns with varying *N_x_*, as a function of the depth coordinate *z*. Specifically, in Figure 4b–e, we report the (*x*, *z*) field intensity distribution. As shown in the figure, the typical decay length rapidly decreases with the pattern resolution, and it becomes vanishingly low for a subwavelength spectral component. Said differently, in the subwavelength regime, the scattered field from a feature decays rapidly along the propagation direction. Since the scattered field is, ultimately, responsible for coupling adjacent planes, we conclude that the spatial coupling between two planes is less prominent for smaller spatial features. The plane–plane coupling is the determining bounding factor in reconstructing adjacent planes.

Figure 4c, corresponding to the case *N_x_* = 1, shows that spatial morphologies of the order of the wavelength decouple on propagations approximately larger than the wavelength. As such, they will affect the information of planes located within this distance. To illustrate this point in more detail, in Figure 4f–j, we report the average spectral intensity as a function of the propagation distance *z* and the sampling ratio *N_x_* for different frequencies. For shorter propagation distances, the vanishing of the intensity does not necessarily mean that coupled planes cannot be reconstructed. On the contrary, it suggests that the full 3D reconstruction of the sample is not easily separable in a series of independent 2D reconstruction problems.

**Figure 5:**
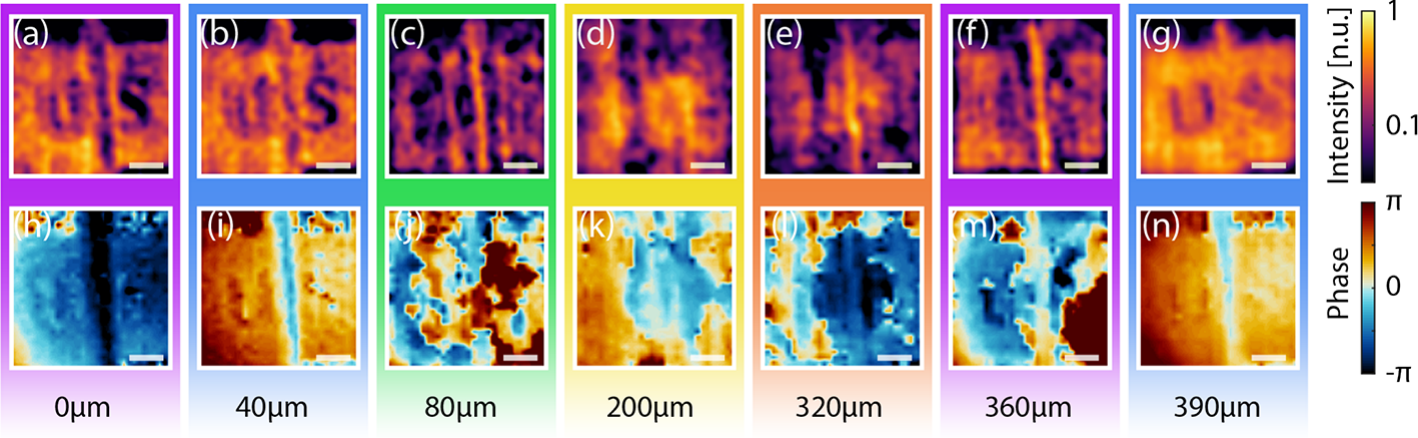
Plane-by-plane volumetric reconstruction of the semitransparent object with subwavelength metallic features presented in Figure 2. (a–g) Intensity profiles averaged between 1.6 and 2 THz. (h–n) Unwrapped phase profiles averaged between 1.6 and 2 THz. The images have been reconstructed by considering different inverse propagation lengths *z*_0_. taken at different depths, respectively, (a, h) *z*_0_ =0 μm, (b, i) *z*_0_ = 40 μm, (c, j) *z*_0_ = 80 μm, (d, k) *z*_0_ = 200 μm, (e, l) *z*_0_ = 320 μm, (f, m) *z*_0_ = 360 μm, and (g, n) *z*_0_ = 390 μm. The white scale bar in all the pictures corresponds to 500 μm.

**Figure 6:**
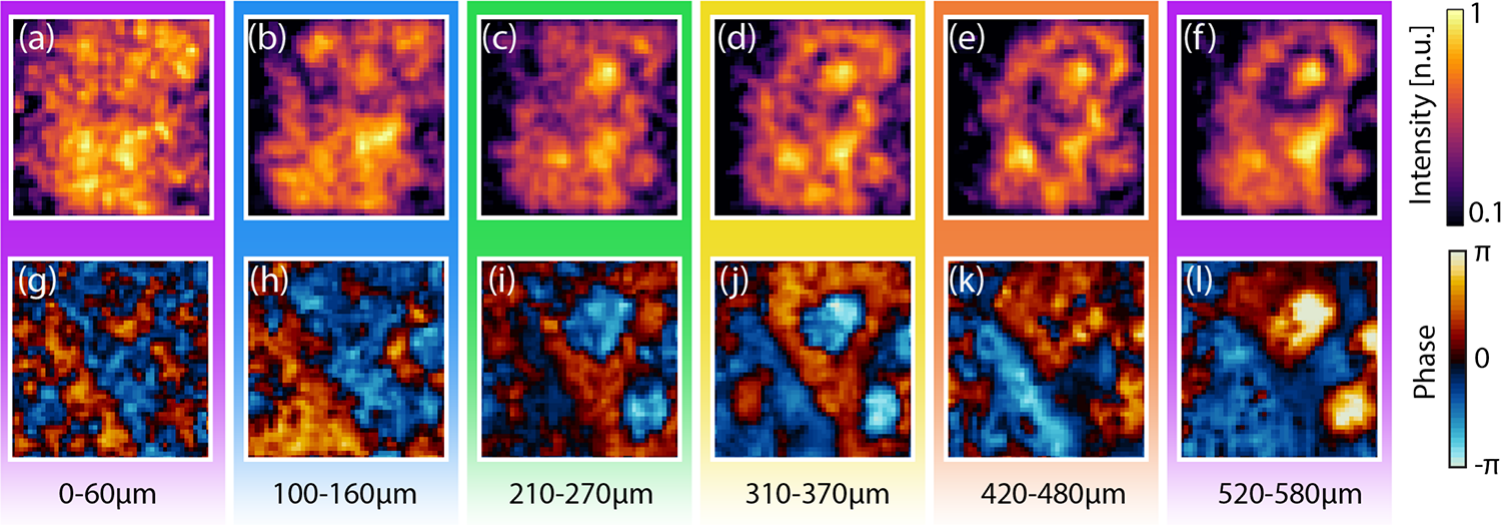
Plane-by-plane volumetric scan along the sample thickness of the sample presented in Figure 3. Spectral and phase images at 0.5 THz averaged between (a, g) *z* = 0 – 60 μm, (b, h) *z* = 100 – 160 μm, (c, i) *z* = 210 – 270 μm, (d, j) *z* = 310 – 370 μm, (e, k) *z* = 420 – 480 μm, and (f, l) *z* = 520 – 580 μm.

In light of this discussion, in Figures 5 and 6, we report a plane-by-plane reconstruction of the experiments performed in the “Results” section. Specifically, Figure 5 corresponds to the full volumetric analysis of the metallic “U” and “S” letters, as obtained through eq 6 by spanning a set of inverse propagation distances *z*_0_. As foreseen through the previous discussion, the visibility of both letters diminishes and mixes for distances corresponding to fractions of the central wavelength, while the air gap between the two letters’ substrates becomes evident for internal propagation distances at the interface between the letters’ substrate and the plastic spacer, e.g., around *z*_0_ = 80 μm and again around *z*_0_ = 360 μm.

Figure 6 reports a similar analysis for the semitransparent sample composed of Teflon and sugar particles (cf. Figure 3). Since the target is mostly transparent, the morphology of the sample becomes particularly easy to discern in the spectral phase domain. For values of *z*_0_ < 160 μm, the features of the sugar and Teflon particles appear mixed and hard to discern. The sugar particles, located at a depth *z* > 500 μm, are relatively larger than the Teflon ones, with an average diameter four times larger than the central wavelength. Consequently, they remain clearly visible even from depths below 200 μm.

The Teflon particles, placed at *z*_0_ > 300 μm and characterized by an average diameter of the order of the wavelength, remain visible only in the phase images between 300 and 600 μm. The vanishing contribution of the Teflon particles for depths *z*_0_ < 270 μm and *z*_0_ > 370 μm strongly hints that imaging behind semitransparent materials could be viable in the subwavelength regime.

## Conclusions

In this work, we investigated the capability of THz TNGI for the field-sensitive reconstruction of 3D, subwavelength morphologies via plane decomposition and inverse propagation of the reconstructed scattered field from the sample. Our unique method uses an inverse propagation approach critically enabled by the ability to detect the full properties of the scattered field. The inverse propagator, based on Green’s function formalism, performs a virtual “refocusing” of the hyperspectral image of the sample, granting access to the spatial and spectral features of internal planes of the sample placed at an arbitrary distance from the THz source, which acts as the probe of our imager.

When the source patterns present deeply subwavelength features, the rapidly varying nature of the electromagnetic Green’s function induces strong spatiotemporal coupling of the field components generated. These microscopic components decay rapidly along the depth dimension, allowing to use of the spatiotemporal coupling effect to separate the different subwavelength contributions of the different elements. We take advantage of this plane-sensitive property to separate and select an arbitrary plane along the propagation axis within microscopic volumes. In particular, we have demonstrated how the inverse propagator allows the retrieval of hidden features not accessible to standard approaches, e.g., time-of-flight imaging.

Our theoretical and experimental details strongly suggest that 3D sparse objects with subwavelength details are particularly suitable for our analysis. Our methodology also shows potential for retrieving partial information of targets placed behind an occluding object, especially in the case of semitransparent materials. These limitations have been thoroughly investigated in different fields, for instance, in hypercentric optics,^14^ and our results provide a concrete starting point for a similar investigation also in time-resolved THz micro-volumetry.

In conclusion, our work makes possible the localization of objects composed of different materials and placed in the interior of complex 3D samples, as long as the object is composed of sparse subwavelength features on planes separated by distances in the order of the wavelength.

In future works, it will be important to investigate the role of denser diffusive media,^43,44^ material transparency, SNR, and complex object geometries in the applicability of our methodology to real-life scenarios. The implementation of additional parallel detection stages for which the propagating light experiences different paths, e.g., by measuring the reflected THz light, would increase the capability to discriminate and isolate planes along the depth dimension. We believe that this demonstration paves the way toward a complete four-dimensional hyperspectral microscopy, i.e., an approach suitable to reveal the THz spectroscopy signatures of any type of morphological features within a microscopic 3D object, having a potential groundbreaking role for the investigation of imaging through complex media, e.g., scatterers.^43−45^

## Supporting Information Available

Vectorial definition of the TNGI for a distant object from the source, experimental setup, sugar and Teflon hyperspectral response, and simulation of refocusing of metallic letters (PDF)
Volumetric reconstruction of a semitransparent object with subwavelength metallic features (AVI)
Temporal reconstruction of a semitransparent object with subwavelength metallic features (AVI)
Volumetric reconstruction of a complex 3D object via refocusing: Teflon and sugar particles on plastic slabs (AVI)
Temporal reconstruction of a complex 3D object via refocusing: Teflon and sugar particles on plastic slabs (AVI)

## Supplementary Material

ph2c01727_si_001.pdf

ph2c01727_si_002.avi

ph2c01727_si_003.avi

ph2c01727_si_004.avi

ph2c01727_si_005.avi

## Notes

The data represented in the paper as deposited as DOI: 10.17028/rd.lboro.21437211.
