# Supplementary material for: Terahertz Nonlinear Ghost Imaging via Plane Decomposition: Toward Near-Field Micro-Volumetry
Source: ACS Photonics. 2023 Mar 10;10(6):1726–34. doi: 10.1021/acsphotonics.2c01727 (PMC10288534; doi:10.1021/acsphotonics.2c01727)
Supplement: Supplementary file 1 — ph2c01727_si_001.pdf [file ph2c01727_si_001.pdf]

Supplementary materials. Terahertz Nonlinear Ghost Imaging via Plane Decomposition: Towards Near-Field Micro-Volumetry

S1. Vectorial definition of the TNGI for a distant object from the source.

Here we briefly recall the theory of TNGI for a distant object from the source, which is at the basis of our volumetric reconstruction. We derive the formalism in terms of the vector potential $\boldsymbol{A}$, to show that an inverse propagator consisting of a simple scalar Green's function fully reconstructs a vectorial problem.[1]

The general Maxwell's equation for the electric field in the presence of a nonlinearly induced current reads as

$$\nabla \times \nabla \times \boldsymbol{E}(\boldsymbol{r},\omega) = \ \omega^2 \epsilon\mu\, \boldsymbol{E}(\boldsymbol{r},\omega) + i\omega\, \mu\, \boldsymbol{J}(\boldsymbol{r},\omega). \tag{1}$$

We define ϵ_0 and μ_o the absolute electric permittivity and magnetic permeability of vacuum, respectively. In an isotropic medium $\epsilon = \epsilon_0\epsilon_r$, $\mu = \mu_0\mu_r$, where $\epsilon_r = 1 + \chi_{el}$ and $\mu_r = 1 + \ \chi_{mag}$ depend on the electrical χ_{el} and magnetical χ_{mag} susceptibility, respectively. In a generic anisotropic medium, these relations take a tensorial form. Furthermore,$c = (1\ /\epsilon\mu)^{1/2}$ is the speed of light and $k_0 = \omega\ c^{-1}$ is the wave vector.

We can introduce the vector potential $\boldsymbol{A}(\boldsymbol{r},\omega)$ and the scalar potential $\phi(\boldsymbol{r},\omega)$ which are related to the electric field $\boldsymbol{E}(\boldsymbol{r},\omega)$ by means of the Lorentz gauge:

$$\mathbf{E}(\boldsymbol{r},\omega) = -i\omega\mu\, \boldsymbol{A}(\boldsymbol{r},\omega) + \mu\nabla\phi(\boldsymbol{r},\omega), \text{ with gauge } \nabla\cdot\boldsymbol{A}(\boldsymbol{r},\omega) = i\omega\ \mu\epsilon\ \phi(\boldsymbol{r},\omega). \tag{2}$$

We define here $\nabla\boldsymbol{A}(\boldsymbol{r},\omega)$ the gradient of $\boldsymbol{A}(\boldsymbol{r},\omega)$ and $\nabla\cdot\boldsymbol{A}(\boldsymbol{r},\omega)$ its divergence. The application of such a gauge leads to the Helmholtz wave equation in the vector potential:

$$\nabla^2\boldsymbol{A}(\boldsymbol{r},\omega) + k_0^2\boldsymbol{A}(\boldsymbol{r},\omega) = \boldsymbol{J}(\boldsymbol{r},\omega). \tag{3}$$

We will use this formalism to model the THz generation from optical conversion which we write explicitly for a nonlinear polarisation source $\boldsymbol{P}_{NL}(\omega)$ such that $\boldsymbol{J}(\omega) = i\ \omega\ \boldsymbol{P}_{NL}\ (\omega)$.

Specifically, considering the nonlinear optical rectification conversion from an optical beam to a THz source, taking place in an infinitely thin plane in $z = 0$, we have $\boldsymbol{P}_{NL}(\omega) = 2\epsilon_0\chi^{(2)}|\boldsymbol{A}^{opt}(\boldsymbol{r},\omega)|^2$.

In the temporal domain, the generated field $\boldsymbol{a}_{\boldsymbol{n}}^{\boldsymbol{THz}}$ is

$$\boldsymbol{a}_n^{THz}(x,y,t) = \partial_t(\boldsymbol{p}_{NL}) = 2\epsilon_0\chi^{(2)}f^{THz}(t)\ I_n(x,y)\ \hat{\boldsymbol{y}}. \tag{4}$$

Here $I(x,y)$ is the intensity of the nth optical pattern converted to THz and $\chi^{(2)}$ a nonlinear conversion coefficient that takes into account the vectorial nonlinear interaction.

[1] The electric field in time defined as $e(t)$, with Fourier transform $\boldsymbol{E}(\omega)$ in the frequency space.

$\mathbf{e}(t) = 2\boldsymbol{Re}\int_{-\infty}^{\infty} \boldsymbol{E}(\omega)e^{-i\omega t}\frac{d\omega}{2\pi}$ Equivalently, we define the spatial Fourier transform in the plane as

$$\boldsymbol{E}(x,y) = \int \check{\boldsymbol{E}}(k_x,k_y) exp(i\, k_x x + ik_y y)\frac{dk_x dk_y}{(2\pi)^2}$$

With no loss of generality, we consider that the interaction produces a source along $\hat{y}$ and we consider $f^{THz}(t) = \left(1 - 2(t/t_0)^2\right)e^{-(t/t_0)^2}$ to be the waveform of an ideal quadratic-rectified optical pulse of duration $t_0 = 357$ fs in our simulations, corresponding to a frequency bandwidth of around 3THz.

To consider the propagation of the field $\boldsymbol{A}(x,y,z_0,\omega)$ at any point of the space, we can use the Green function of the Helmholtz equation, which, for a planar source in $z = 0$, we define as $G(x,y,z_0,\omega)$. Explicitly referring to our methodology, considering the nth pattern of our set $I_n(x,y)$, the distribution of $\boldsymbol{A}(x,y,z_0,\omega)$ in the plane z_0, where the object is, can be expressed as

$$\boldsymbol{A}_n^{THz}(x,y,z_0,\omega) = \int \frac{\check{\boldsymbol{J}}(\mathbf{k},\omega)}{k_0^2 - |\boldsymbol{k}|^2}\exp\,(i\,\mathbf{k}\cdot\mathbf{r})\frac{d\boldsymbol{k}}{(2\pi)^3} \tag{4}$$

$$=2\epsilon_0\chi^{(2)}F^{THz}(\omega)I_n^P(x,y,z_0)\,\hat{y},$$

where $I_n^P(x,y,z_0)$ is the propagated pattern

$$\boldsymbol{I}_n^P(x,y,z_0) = \boldsymbol{I}_n(x,y,z=0) * \boldsymbol{G}(x,y,z_0,\omega), \tag{5}$$

where the convolution is intended in the transverse coordinates (x,y).

The spectral and morphological features of such a sample are represented with the complex-valued transmission function $\boldsymbol{M}(x,y,z_0,\omega)$, as discussed in the main text. Because the electric field $\boldsymbol{E}(\boldsymbol{r},\omega)$ and the vector field $\boldsymbol{A}(\boldsymbol{r},\omega)$ are related by a linear operator, $\boldsymbol{M}(x,y,z_0,\omega)$ transforms, in the same way, both $\mathbf{E}(\boldsymbol{r},\omega)$ and $\mathbf{A}(\boldsymbol{r},\omega)$. After the interaction with the object, then, the vector potential is

$$\boldsymbol{A}_{\boldsymbol{n}}^{\boldsymbol{obj}}(x,y,z_0\omega) = \boldsymbol{M}(x,y,z_0,\omega)\boldsymbol{A}_{\boldsymbol{n}}(x,y,z_0,\omega). \tag{6}$$

The TDS detection in the Fourier plane, as described in our previous papers [1–3], selects the wavevector at $k_x = 0$ and $k_y = 0$ of the electric field at a given polarisation, which is directly proportional to the average of $\boldsymbol{A}_{\boldsymbol{n}}^{\boldsymbol{obj}}$ by

$$\check{\boldsymbol{E}}_{\boldsymbol{n}}^{\boldsymbol{obj}}(k_x = 0,k_y = 0,z = z_0) \propto \check{\boldsymbol{A}}_{\boldsymbol{n}}^{\boldsymbol{obj}}(k_x = 0,k_y = 0,z = z_0). \tag{7}$$

For a detection along the polarisation parallel to the y axis, we then have a coefficient defined as:

$$C_n(\omega) = \iint \boldsymbol{A}_n^{obj}(x,y,z_0,\omega)dxdy = \eta_0^2\chi^{(2)}F(\omega)\iint \boldsymbol{I}_n^P(x,y,z_0)\,\boldsymbol{T}(x,y,z_0,\omega)dxdy$$
$$= \eta_0^2\chi^{(2)}\,F(\omega)\iint \check{\boldsymbol{I}}_n^P(k_x,k_y,z_0)\,\check{\boldsymbol{T}}(k_x,k_y,z_0,\omega)\frac{dk_xdk_y}{(2\pi)^2}\,|_{k_y,k_y=0}\,. \tag{8}$$

This coefficient contains the full electromagnetic information of our problem and represents a scalar product between the object $\boldsymbol{M}$ and the $\boldsymbol{I}_n^P$ both in the direct and Fourier space. It is particularly convenient to express the reconstruction in the Fourier space. The TNGI reconstructed transmission function $\check{\boldsymbol{M}}^{GI}$ can be then expressed as

$$\check{\boldsymbol{M}}^{GI}(k_x,k_y,\omega) = \sum\nolimits_n C_n(\omega)\check{\boldsymbol{I}}_n\,(k_x,k_y) - \sum\nolimits_n C_n(\omega)\sum\nolimits_n\iint \check{\boldsymbol{I}}_n\,(k_x,k_y)\frac{dk_xdk_y}{(2\pi)^2} \tag{9}$$

By recalling that the pattern functions belong to an orthogonal set, and by noticing that $C_n(\omega)$ can be equivalently expressed as

$$C_n(\omega) = 2\epsilon_0\chi^{(2)}F^{THz}(\omega)\iint \check{\boldsymbol{I}}_n\,(k_x,k_y)\,\check{\boldsymbol{G}}(k_x,k_y,z_0,\omega)\,\check{\boldsymbol{M}}(k_x,k_y,z_0,\omega)\frac{dk_xdk_y}{(2\pi)^2}, \tag{10}$$

the reconstructed transmission function is expressed as

$$\check{\boldsymbol{M}}^{\boldsymbol{GI}}(k_x,k_y,\omega) = \check{\boldsymbol{G}}(k_x,k_y,z_0,\omega)\,\check{\boldsymbol{M}}(k_x,k_y,z_0,\omega), \tag{11}$$

and the ground-truth 'inverse-propagated' transmission function $\check{\boldsymbol{M}}^{IP}$ is obtained with the operation

$$\check{\boldsymbol{M}}^{IP}(k_x,k_y,z_0,\omega) = \check{\boldsymbol{W}}(k_x,k_y,z_0,\omega)\check{\boldsymbol{M}}^{GI}(k_x,k_y,\omega) = \frac{\check{\boldsymbol{M}}^{GI}(k_x,k_y,\omega)}{\check{\boldsymbol{G}}(k_x,k_y,z_0,\omega)}. \tag{12}$$

Now, the Green function of a source on the plane $z = 0$ for the Helmholtz equation is simply

$$\check{\boldsymbol{G}}(\boldsymbol{k},\omega) = \frac{1}{k_0^2 - |\boldsymbol{k}|^2}\exp(i\boldsymbol{k}\cdot\boldsymbol{r} + i\omega t) \tag{13}$$

where $\boldsymbol{k} = \left(k_x,k_y,\frac{2\pi}{z_0}\right)$ or alternatively,

$$\boldsymbol{g}(\boldsymbol{r},t) = \frac{v}{\sqrt{4\pi\,|\boldsymbol{r}|}}\delta(|\boldsymbol{r}| - vt)\,\theta(t) \tag{14}$$

here v is the speed of light inside the medium, δ is the Dirac's function and θ is the Heaviside step function. This scalar function can be explicitly used to solve the full vectorial electromagnetic problem and reconstruct the object function.

S2. Experimental setup

For completeness purposes, we report in Fig. S1 the experimental configuration. Additional details can be found in the supplementary information of [2]. A femtosecond source is split into two beams, i.e. a pump and a reference beam. The pump is shaped by a digital micromirror array device (DMD) spatial light modulator, and the optical patterns are used to illuminate a ZnTe crystal for terahertz generation via optical rectification. The sample is placed in close proximity to the nonlinear crystal, and the THz light transmitted is collected by a single parabolic mirror (PM) for a Fourier single-spatial-frequency detection [2].

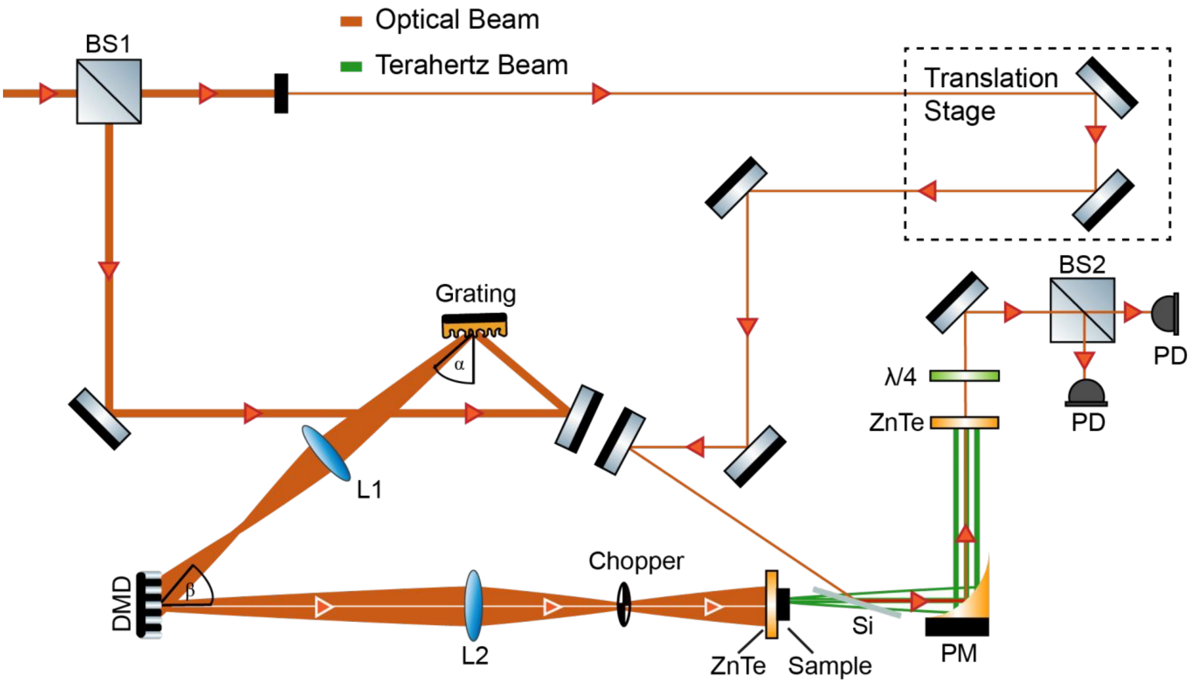

Fig. S1: Experimental scheme of the Time-resolved Nonlinear Ghost Imaging setup. Here $\alpha = 26.3°$ and $\beta = 71.3°$, beam splitter (BS), photodetector (PD), silicon film (Si), parabolic mirror (PM).

S3. Sugar and Teflon hyperspectral response

We here show the spectral and temporal response of Teflon and sugar particles placed close to the generation crystal surface, Fig. S1 and Fig. S2 respectively. As mentioned in the main text, the Teflon has a strong phase response while the morphology is not easily retrievable from the amplitude images as it is a poor absorber and a weakly scattering medium. The sugar, on the contrary, has higher absorption around 2 THz, and a strong scattering from the edges below 1 THz.

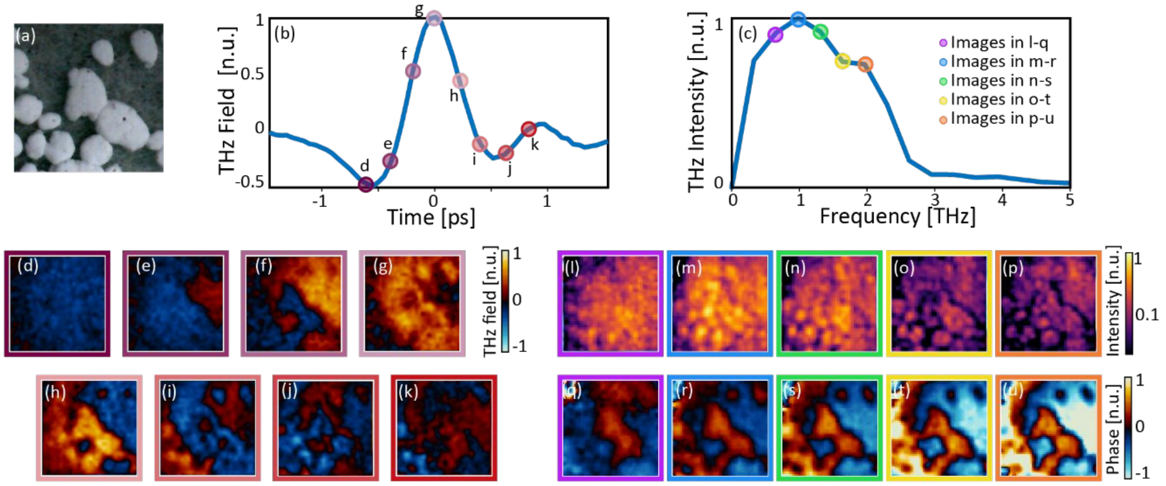

Fig. S2: Temporal and Hyperspectral response of particles of Teflon. (a) Optical image of the sample. (b) TDS acquisition. (c) Spectrum (d-k) Temporal slices are indicated shown in (b). (l-p) Hyperspectral images at different frequencies. (q-u) Phase response at the frequencies shown in (c). In all panels, the field of view is 4 mm x 4 mm with a 32 x 32 spatial sampling.

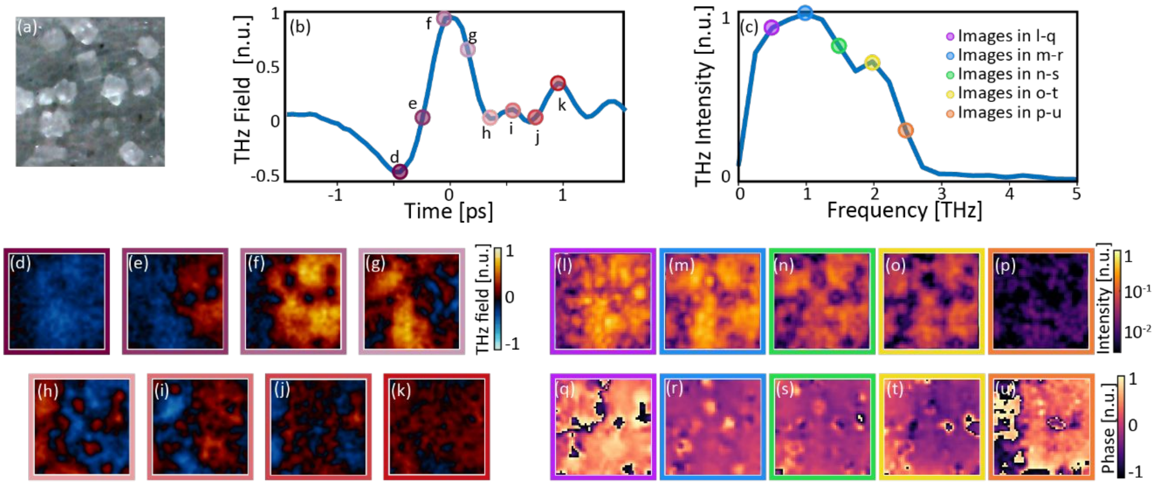

Fig. S3: Temporal and Hyperspectral response of particles of Sugar. (a) Optical image of the sample. (b) TDS acquisition. (c) Spectrum (d-k) Temporal slices are indicated shown in (b). (l-p) Hyperspectral images at different frequencies. (q-u) Phase response at the frequencies shown in (c). In all panels, the field of view is 4 mm x 4 mm with a 32 x 32 spatial sampling.

S4. Simulation of refocusing of metallic letters

Reproducing the experimental scenario of Fig. 2, we simulated the imaging process of an object comprising two completely opaque masks placed at different depths ($z = 0$ and $z = 300\ \mu m$) in a 3-dimensional volume filled with a transparent medium. The result represents a 2 mm x 2 mm field-of-view with a spatial sampling of 32 x 32 pixels, matching the experimental conditions. The letter 'S' and 'U' are placed at the wavelength distance. Every pixel is about $62\ \mu m$ and the letters cover an area of about $800 \times 600\ \mu m$, about twice the wavelength, with, however, much smaller features. Comparing these dimensions with the Green function propagation summary in Fig. 5, we expect that the letter 'U' loses visibility on the plane of the letter 'S'. Indeed, Fig 2a,b shows that the mask at a negligible distance from the source (z=0), representing the letter 'S' appears visible in spectral images

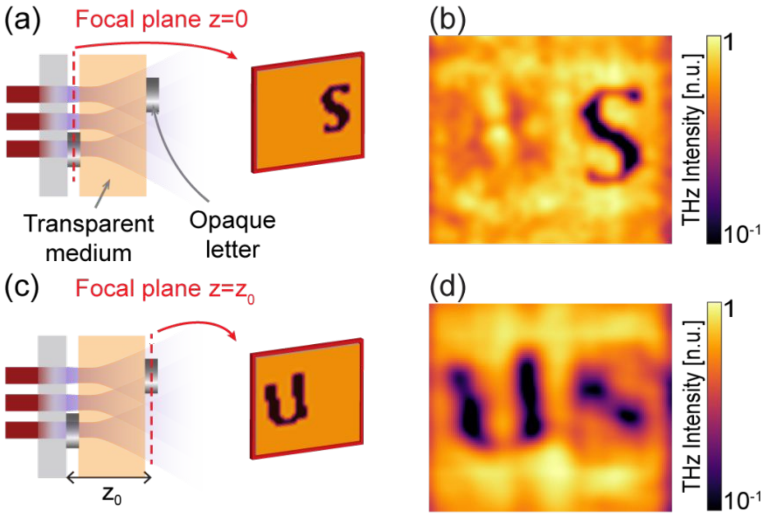

Fig. S4: Simulated volumetric reconstruction of a semi-transparent object with subwavelength opaque features. (a) Conceptual illustration of the imaging scheme: the refocusing plane is placed at z=0µm. (b) Simulated hyperspectral image averaged between 0.8 and 1.2 THz. (c) Conceptual illustration: the refocusing plane is placed at z0=300 µm. (d) Simulated image at the focal plane z=300µm, averaged between 0.8 and 1.2 THz. In all panels, the simulated field of view was 2 mm x 2 mm with a 32x32 spatial sampling refocused image

(represented by a portion of the spectrum centered at 1 THz). The second mask, representing the letter 'U' is negligibly visible and distorted. Similarly, the letter 'U' can be refocused by applying Eq. 6. As already discussed, the letter 'S' loses visibility but still absorbs the imaging field. Such simulations confirm the experimental findings in Fig. 2 and Fig.5.

References

(1) Olivieri, L.; Totero Gongora, J. S.; Pasquazi, A.; Peccianti, M. Time-Resolved Nonlinear Ghost Imaging. *ACS Photonics* **2018**, *5* (8), 3379–3388. https://doi.org/10.1021/acsphotonics.8b00653.

(2) Olivieri, L.; Gongora, J. S. T.; Peters, L.; Cecconi, V.; Cutrona, A.; Tunesi, J.; Tucker, R.; Pasquazi, A.; Peccianti, M. Hyperspectral Terahertz Microscopy via Nonlinear Ghost Imaging. *Optica* **2020**, *7* (2), 186. https://doi.org/10.1364/OPTICA.381035.

(3) Totero Gongora, J. S.; Olivieri, L.; Peters, L.; Tunesi, J.; Cecconi, V.; Cutrona, A.; Tucker, R.; Kumar, V.; Pasquazi, A.; Peccianti, M. Route to Intelligent Imaging Reconstruction via Terahertz Nonlinear Ghost Imaging. *Micromachines* **2020**, *11* (5), 521. https://doi.org/10.3390/mi11050521.
